# The Swedish version of the Athletic Fear Avoidance Questionnaire: a psychometric study conducted among amateur male and female soccer players

**DOI:** 10.1186/s13102-026-01769-8

**Published:** 2026-06-08

**Authors:** Kristin Byström, John Ressman, Eva Rasmussen-Barr

**Affiliations:** Department of Neurobiology, Care Sciences and Society, Division of Physiotherapy, Karolinska Institutet, Alfred Nobels Allé 23, Huddinge, 141 83 Sweden

**Keywords:** AFAQ, Fear-avoidance, Kinesiophobia, Return to play, Re-injury

## Abstract

**Background:**

Given the established merits and psychometric properties of the Athletic Fear Avoidance Questionnaire (AFAQ), the present study aimed to translate, cross-culturally adapt and validate the tool for use in a Swedish context.

**Methods:**

We included 95 amateur soccer players (male *n* = 31, female *n* = 64) from a convenience sample of eight soccer teams, who reported a sports injury within the past 12 months and were older than 15 years (mean 22, SD ± 6). They answered web-based surveys (SurveyMonkey©), including the AFAQ and the Tampa Scale for Kinesiophobia (TSK). Construct validity of the AFAQ was evaluated relative to the TSK, using Spearman’s Rho. Reliability was assessed in a subset of participants (*n* = 53) with a test-retest evaluation of the individual items (Kappa^W^) and the total score (Intra Class Correlation, ICC_2.1_). Internal consistency of the tool was assessed through Cronbach’s Alpha (α).

**Results:**

The cross-cultural translation and adaptation of the AFAQ was considered sound. The AFAQ-Swe showed a strong correlation with the TSK (*r* = 0.82), in line with the predefined hypothesis. The test-retest results demonstrated good-to-substantial reliability for the individual items (Kappa^W^ 0.52–0.82) and excellent reliability for the total score (ICC_2.1_= 0.80). The internal consistency was considered satisfactory (α = 0.85).

**Conclusions:**

The AFAQ-Swe demonstrates good measurement properties, with strong reliability and validity. The AFAQ-Swe is a concise, user-friendly tool that can be effectively utilised in the Swedish context, particularly among soccer players. However, further research is necessary to evaluate AFAQ’s predictive validity for a safe return to sports.

## Introduction

Soccer is the most popular sport globally, boasting more than 265 million players at different levels [1]. Due to the nature of the sport and the large number of participants at the elite and amateur levels, the absolute number of injuries is high [2–4]. Sonesson et al. (2023) reported that any given week, almost one in five male and one in four female amateur soccer players reported new or ongoing injuries [4]. Professional players are expected to sustain two injuries per season, anticipating around 50 injuries in a full team [2].

Rehabilitation in sports medicine has typically taken a biomechanical approach, linking body mechanics to athletic performance and the ability to meet the sport’s demands [5, 6]. As a result, many return-to-sport criteria emphasise tests to assess the physical skills required for the sport [7–9]. However, effectively reducing re-injury risk and supporting a safe return to sport requires a holistic perspective that also considers psychosocial factors [6, 7, 10]. Despite this, most rehabilitation research remains focused on physical treatments with limited attention to psychological aspects [11, 12]. Factors such as fear-avoidance and lower self-efficacy have been highlighted as important in ensuring a safe return to sports [11]. The literature indicates that fear avoidance is significantly linked to chronic pain and plays a crucial role in the rehabilitation of musculoskeletal injuries [13, 14]. Kvist & Silbernagel (2022) stated that an athlete’s fear can be categorised into three components: fear of reinjury, fear of enduring lifelong debilitating pain, and fear of being unable to return to sport [5]. It is also suggested that low fear avoidance is associated with a higher likelihood of returning to pre-injury levels of participation and with a quicker return to sport [15]. The impact of fear of reinjury may thus consequently lead to poor rehabilitation outcomes and thus negatively affect the ability to return to sport or be associated with a less favourable recovery [16, 17].

The fear-avoidance model (FAM) is a psychological model that posits that emotional reactions to pain and a high level of fear avoidance can lead to dysfunction [18]. The model includes 4 components: fear of pain, kinesiophobia, fear-avoidance belief, and catastrophizing [18]. Kinesiophobia is an excessive, irrational, and debilitating fear of physical movement and activity, usually resulting from a fear of re-injury or pain [19]. There are several tools for measuring fear-avoidance, and the gold standard questionnaire is the Tampa Scale for Kinesiophobia (TSK) [19]. Other commonly used tools include the Fear-Avoidance Beliefs Questionnaire (FABQ) [20, 21] and the Pain Catastrophizing Scale (PCS) [22]. These tools, however, were developed for a general population and not specifically to measure fear-avoidance among athletes. Due to the lack of assessment tools for measuring fear-avoidance among athletes, the Athletic Fear Avoidance Questionnaire (AFAQ) was developed in 2015 to measure injury-related fear avoidance [23]. Additionally, the Injury Psychological Readiness to Return to Sport Scale (I-PRRS) was developed for use at the end of a rehabilitation period [24]. In contrast, the AFAQ is intended for use at the beginning of rehabilitation to identify any psychological obstacles the athlete may be facing, thereby optimising rehabilitation [23]. By identifying athletes with high levels of fear avoidance using a sport-specific scale, clinicians can address these psychological challenges early in the rehabilitation process, potentially reducing the time it takes for the athlete to return to play.

The AFAQ has to date been cross-culturally translated, adapted, and validated into several languages, including Arabic [25], Brazilian [26], German [27] and Italian [28]. The AFAQ has, in addition, been examined for predictive validity in a cohort of athletes, comparing the AFAQ outcomes with those of the TSK and the FABQ, and with the AFAQ showing a strong correlation with the rehabilitation period required for a safe return to sport [29]. Currently, no validated tool in Swedish is available to assess fear avoidance and fear of reinjury in athletes. As we believe that the AFAQ is beneficial for clinicians as well as for researchers in the sports medicine context in Sweden, the present study aimed to translate, cross-culturally adapt and validate the tool by assessing its face and construct validity, test-retest reliability, and internal consistency.

## Methods

### Design

The study was conducted in two steps: the first step involved a cross-cultural adaptation of the AFAQ from English to Swedish in accordance with international guidelines [30]. The second step involved investigating the AFAQ’s psychometric properties, evaluating the instrument’s face and construct validity, and assessing its test-retest reliability and internal consistency. The study was approved by the Regional Ethical Review Board in Stockholm and Karolinska Institutet, to which ethical approval belongs (Dnr: 2024-06-24, 2024-04318-02-597083).

### Study population

The sample size for the study was determined based on recommended quality standards for assessing the measurement properties of instruments, which suggest a minimum of 50 to 100 participants for evaluating test-retest reliability and 100 or more participants for evaluating construct validity [31].

Participants were recruited by mailing study information to a convenience sample of 13 soccer teams in Sweden. Five teams responded and were interested in participating. In addition, information about the study was posted in an online network group for physiotherapists supporting soccer teams in Sweden, leading to contact with three additional soccer teams. Soccer players who met the eligibility criteria were provided with information about the study through a text message, including a link to the survey (SurveyMonkey©). By completing and submitting the online survey, the participants were considered to have given their informed consent to participate in the study. The inclusion criteria specified that participants must be active soccer players in a team, be 15 years or older, and have experienced a sports injury or musculoskeletal pain within the past 12 months. Conversely, the exclusion criteria included an inability to understand spoken and written Swedish.

### Translation and cross-cultural adaptation

#### The athletic fear avoidance questionnaire (AFAQ)

The AFAQ is a self-report questionnaire comprising 10 statements about the athlete’s feelings and thoughts regarding their injury. Each statement is rated from 1 to 5, where 1 represents “not at all” and 5 represents “completely agree” [23]. The total score ranges from 10 to 50 points, with higher scores indicating greater fear of movement and lower scores suggesting less fear or none at all. The original instrument is considered to have good internal and external validity for measuring fear of movement related to sports injuries [23].

The original AFAQ was validated through a cross-cultural adaptation process based on international guidelines [30].

1. Two native Swedish translators fluent in the English language each translated the original AFAQ from English to Swedish: one an expert (an advanced physiotherapist and researcher in musculoskeletal health and sports medicine), and the other naive. A synthesis of the two translations was prepared, and this version was compared with a previously translated AFAQ to control for consistency. Minor changes to the wording were made, resulting in a preliminary Swedish version (A1).
2. The preliminary Swedish version (A1) was back-translated into English by two naive translators knowledgeable in both Swedish and English, resulting in an English version (A2).
3. The expert group discussed both versions (A1 and A2) and reached a consensus on a pre-final Swedish version, hereafter called the AFAQ-Swe. The expert group comprised three physiotherapists: one advanced physiotherapist in Sports Medicine, one advanced physiotherapist and researcher in Sports Medicine and one advanced physiotherapist and researcher in musculoskeletal health.

### Test of the pre-final version of the AFAQ-Swe

Fifteen active soccer players were included to assess the face validity of AFAQ-Swe. During one-on-one meetings with a physiotherapist, participants were encouraged to share their thoughts on the items in the AFAQ-Swe as they completed the tool. The physiotherapist prompted them to share any difficulties they had in understanding the questions, especially if they found any wording unclear or challenging. The participants were invited to suggest any changes they felt were necessary [32].

### Data collection

The first survey (SurveyMonkey©) was sent to 206 participants and comprised questions on demographic and players’ data, the AFAQ-Swe, and the TSK as a reference instrument. The TSK was chosen since it is considered to measure the same or a similar construct as the AFAQ [14]. The TSK comprises 17 items in the original version and was developed to measure kinesiophobia [19]. Every item is rated on a Likert scale from 1 to 4, where 1 is “strongly disagree” and 4 is “strongly agree” with a total score varying from 17 to 68 points. A value of more than 37 points, with a measurement error of 3 points, indicates kinesiophobia. The Swedish version of the TSK is considered valid and reliable [33].

### Floor and ceiling effects

Floor (best status) and ceiling (worst status) effects were evaluated and considered to be present if more than 15% of the participants reported the highest or the lowest possible score [34].

### Test-retest reliability

We investigated test-retest reliability with an interval of 7–10 days, as previously recommended [31, 35]. The second web-based survey (SurveyMonkey©) was sent to sixty-five consecutive participants, also included in the validity part of the study, and included the AFAQ-Swe and a question on global change to detect whether the participant had experienced a new injury or worsening of an ongoing injury since answering the first survey. The question about change was answered with “worse,” “not changed,” or “improved.” Those who answered “worse” were excluded from the test-retest analysis. For non-responders to the second survey, a reminder was sent after three days and again after three days.

To analyse the test-retest reliability of the total score of the AFAQ-Swe, the Intra Class Correlation (ICC _2.1)_ was used with a two-way random effects model [31, 36]. The ICC has a range from 0 to 1, where 0.60–0.80 is regarded as good and > 0.80 is regarded as excellent [31, 36]. To assess the degree of agreement for each separate item included in the AFAQ-Swe, a weighted Cohen’s Kappa-coefficient (K^w^) was used [37]. Kappa was interpreted as κ < 0.1 = poor; κ: 0.1– 0.2 = slight; κ: 0.21–0.40 = fair; κ: 0.41–0.6 = moderate; κ: 0.61–0.8 = substantial and κ: 0.81–1 = almost perfect [37].

### Internal consistency

Cronbach’s alpha (α) was used to calculate the internal consistency among the items of the AFAQ-Swe. Internal consistency measures the degree of interrelatedness among the items [38]. A low Cronbach’s α represents a lack of correlation among the items of the instrument, whilst an α coefficient equal to or greater than 0.70 is considered sufficient evidence for internal consistency [38]. A correlation analysis was conducted to investigate the association between the AFAQ-Swe’s total score and each item using Spearman’s Rank correlation coefficient (rho).

### Construct validity

Construct validity is defined as “*the degree to which the scores of an instrument are consistent in relation to predefined hypotheses based on the assumption that the instrument validly measures the construct that is to be measured*” [31]. Construct validity was assessed by comparing the AFAQ-Swe to the TSK since the TSK tool is considered the “golden standard” for measuring kinesiophobia or fear avoidance [14]. A priori, a hypothesis was set to *r* > 0.3, which is considered a moderate correlation, since values over 0.3 are acceptable [35]. The coefficients are described as low (< 0.3), moderate (< 0.3–0.6) and high (> 0.6) [35]. Spearman’s Rank correlation coefficient (Rho) was used to analyse the association between the AFAQ-Swe and the reference scale.

## Results

Table 1 shows the demographic data and the results of the AFAQ-Swe and the reference instrument. A total of ninety-five consecutive participants were included in the validity part of the study, 31 men and 64 women, with a mean age of 22 (SD ± 6, range 15–39). They reported 14 active years (SD ± 4.7) in soccer play and a weekly soccer practice of 7,5 h (SD 2.9). Fifty-three (*n* = 53) participants were included in the test-retest part of the study.

**Table 1:** Baseline demographic data and sports characteristics for the participants in the validity part of the study (*n* = 95) and in the reliability part of the study (*n* = 53)

	All participants *n* = 95	Test-retest *n* = 53
Age, yr (mean, SD)	22 (± 6)	21 (± 5)
Sex		
Male (n, %)	31 (33)	14 (26)
Female (n, %)	64 (67)	39 (74)
Active years in soccer (mean, SD)	14 (± 4.7)	13 (± 4.2)
Weekly hours of soccer practice (mean, SD)	7.5 (± 2.9)	7.6 (± 3.0)
Total number of injuries, past 12 months (n, %)		
Knee	32 (33.3)	18 (33.9)
Foot	23 (23.9)	12 (22.6)
Back	4 (4.1)	2 (3.8)
Hip	7 (7.3)	7 (13.2)
Shoulder	4 (4.1)	1 (1.8)
Neck	1 (1.0)	1 (1.8)
Other	27 (28.1)	14 (26.4)
AFAQ-Swe score (score 10–50), (mean, SD) Tampa (score 17–68), (mean, SD)	20.6 (± 7.7) 34.1 (± 6.9)	21.6 (± 7.7) 34.6 (± 6.5)

*AFAQ* Athletic Fear Avoidance Questionnaire, *SD* standard deviation (±)

### Translation and face validity

The AFAQ was successfully translated from English into Swedish, yielding the AFAQ-Swe, which was consistent with a previous translation (unpublished data) of the tool. Testing the pre-final version revealed minor changes that did not affect the final version. The participants expressed no difficulty understanding the questions or the wording.

### Floor and ceiling effects

The floor effect for the AFAQ-Swe was 9.5%, with 9 of 95 participants (*n* = 9) scoring the lowest total score. None of the participants scored highest, and therefore, there was a 0% ceiling effect.

### Test-retest reliability

Fifty-five participants completed the second survey, and two reported “worse” on the global change question, indicating increased pain or a new injury, and were therefore omitted from the analysis. The analyses of test-retest reliability were thus based on fifty-three participants (*n* = 53). The mean Kappa-score ranged from 0.55 (moderate) to 0.82 (substantial), with a median of 0.62 (Table 2). The test-retest reliability of the AFAQ-Swe total score was 0.80 (ICC_2.1_).

**Table 2:** Test-retest reliability (*n* = 51) of the Swedish version of the AFAQ total score (10–50) and the ten separate items

Item	Kappa^W^	CI 95%	PA%	CI 95%	SEM
1. Jag kommer aldrig att kunna spela som jag gjorde innan min skada. *I will never be able to play as I did before my injury.*	0.57	0.21–0.93	63	48–76	0.18
2. Jag är orolig för att min roll i laget ska förändras. *I am worried about my role with the team changing.*	0.63	0.40–0.86	51	37–65	0.12
3. Jag är orolig för vad andra ska tycka om mig om jag inte kan spela/träna på samma nivå. *I am worried about what other people will think of me if I don’t perform at the same level.*	0.61	0.31–0.91	57	42–70	0.15
4. Jag är inte säker på vilken skada jag har. *I am not sure what my injury is.*	0.62	0.28–0.95	67	52–79	0.17
5. Jag tror att min nuvarande skada har riskerat min framtida atletiska/idrottsförmåga. *I believe that my current injury has jeopardised my future athletic abilities.*	0.69	0.49–0.90	55	40–69	0.11
6. Jag är inte bekväm med att gå tillbaka till min träning förrän jag är 100%. *I am not comfortable going back to play until I am 100%.*	0.60	0.33–0.87	55	40–69	0.14
7. Folk förstår inte hur allvarlig min skada är. *People don’t understand how serious my injury is.*	0.82	0.79–0.84	65	50–77	0.01
8. Jag vet inte om jag är redo att spela eller träna igen. *I don’t know if I am ready to play.*	0.68	0.47–0.88	61	46–73	0.10
9. Jag är orolig för att om jag går tillbaka och tränar för snart igen så kommer det att göra min skada värre. *I worry that if I go back to play too soon*,* I will make my injury worse.*	0.55	0.21–0.89	49	35–63	0.17
10. När smärtan är intensiv är jag orolig för att min skada är väldigt allvarlig. *When my pain is intense*,* I worry that my injury is a very serious one.*	0.82	0.68–0.90	55	40–69	0.06
Total score AFAQ*	0.80	0.68–0.88			

*Analysed with ICC_2.1_,

*PA* Proportion of Agreement, *CI* Confidence Interval, *SEM*  Standard error of measurement

### Internal consistency

Cronbach’s α reached 0.85, which is satisfactory. Additional analysis of Cronbach’s α using an “if item deleted analysis” showed a minor increase to 0.86 if item 4 (*I am not sure what my injury is)* was deleted. Removal of any other item indicated a lower internal consistency. There was a significant correlation between the separate items and the AFAQ-Swe total score.

### Construct validity

The correlation between AFAQ-Swe and the reference instrument, the TSK, was high (*r* = 0.82), confirming the a priori hypothesis (*r* > 0.30).

## Discussion

We aimed to translate and adapt the original AFAQ into Swedish cross-culturally and to investigate its reliability and certain aspects of validity. Our study showed that the AFAQ-Swe version has acceptable psychometric properties that concur with the previously conducted cross-cultural validation studies of the tool [23, 25–28].

The AFAQ is proposed to assess the construct of fear avoidance, among injured athletes, an important psychological factor to consider, for a safe return to sport or play [5, 15, 23]. The unidimensional nature of the AFAQ, measuring the construct of fear avoidance, was confirmed in the original study [23] and in the Brazilian [26] and Italian validation studies [28]. Nonetheless, the Italian validation study suggested that the AFAQ might be considered a formative tool, measuring additional constructs, as its included items address slightly different factors beyond fear-avoidance [28]. Still, considering that the fear-avoidance model comprises fear of pain, kinesiophobia, fear-avoidance beliefs, and catastrophizing, which cover the content of the AFAQ, this might be debated [18]. Our study found acceptable internal consistency (α = 0.85) for the AFAQ-Swe, which is in line with previous validation studies [23, 25–28]. The value of α shows how well the items proposed to measure the same general construct in an instrument correspond to each other and to the total score [38]. Based on our results of the internal consistency, we concluded that the individual items in the AFAQ-Swe effectively measure the construct of fear avoidance related to sports and re-injury. As such, we decided not to conduct a factor analysis. A future study, however, might consider using a modern test theory, such as the Rasch model, to further assess the unidimensional nature of the AFAQ [39].

We investigated the test-retest reliability of the total score of the AFAQ-Swe, showing an excellent reliability (*r* = 0.80) in accordance with the previous validation studies, reporting an ICC between 0.79 and 0.95 [23, 25–28]. In addition, we assessed the AFAQ-Swe’s separate items for test-retest reliability, as the reliability of the included items can affect the ICC estimate, which depends on sample variance [40]. Even if the overall ICC is considered acceptable, some items may show lower test-retest reliability, suggesting difficulty with the wording or understanding of included items. To our knowledge, none of the previous validation studies assessed or reported test-retest reliability for the specific items of the AFAQ, which we consider a limitation. Although the separate items in the AFAQ-Swe showed good to substantial reliability, the Kappa values still differed somewhat and might have affected the ICC estimate. The item seven [7] “*When my pain is intense*,* I worry that my injury is a very serious one*” showed the highest Kappa value (0.82) with a narrow 95% CI and a small SEM, while the item one [1] “*I will never be able to play as I did before my injury”* showed a lower Kappa value (0.57) with a larger 95% CI and in addition a larger SEM meaning a somewhat lower reliability. Although the total score of the AFAQ-Swe indicates the athlete’s overall level of fear avoidance, the separate items can support the trainer or the physiotherapist to better understand what type of fear avoidance or fear of reinjury is important to consider in the rehabilitation process. Hence, the answers to the separate questions might be used in the dialogue with the athlete.

The strengths of our study include adherence to international recommendations, conducting a cross-cultural adaptation and validation study, strong test-retest reliability, and a thorough validation process. We also assessed construct validity using the COSMIN taxonomy based on hypothesis testing [38]. Still, several limitations need to be considered. Based on COSMIN recommendations, we recruited the recommended number of participants for the validity and reliability phase of the study [31, 38]. We initially sent the first survey to 206 eligible participants in the validation phase, but only 95 responded. This number is comparatively low, particularly when contrasted with the Italian and Brazilian validation studies, which involved over 100 participants each [26, 28]. A larger sample size might have provided more robustness to our results. Despite the lower number of participants, our results align with previous validation studies [23, 25–28].

We utilised a single reference instrument to assess the construct validity of the tool and found a high correlation between the AFAQ-Swe and the TSK, supporting our initial hypothesis. It can be debated and seen as a limitation that we relied solely on a single reference instrument to evaluate the construct validity. Nevertheless, given that the TSK is recognised as a “gold standard” for measuring fear-avoidant behaviour, we believe it was a valid choice [19]. We also had no a priori hypothesis for comparing the AFAQ-Swe with other constructs, in contrast with previous validation studies of the AFAQ. They used reference instruments for pain [25, 26, 28], catastrophizing [26, 28], and depression and anxiety [26], reporting correlations ranging from low to moderate. Most of the previous validation studies used the FABQ to analyse construct validity of the AFAQ fear, reporting low to moderate correlations. Only the German validation study used the TSK and reported a good correlation (*r* = 0.57), although lower than ours [27].

Previous validation studies, as well as the original study, included a variety of athletes, among others, soccer players [23, 25–28]. Our study focused on soccer players, thus a single sport, which may limit the generalizability of our results to other sports. In addition, our participants were soccer players at an amateur level. The AFAQ-Swe was, however, recently used in a study of elite women soccer players evaluating the association of various factors with the outcome of the single-leg squat test [8].

Finally, to be eligible for our study, participants reported experiencing musculoskeletal injury or pain in the last 12 months. We, however, do not have data on the severity of the participants’ injuries and whether this might have affected their responses to the AFAQ-Swe.

According to COSMIN, several aspects of validity must be examined for an instrument to be deemed sufficiently valid [31]. Our study did not assess the predictive validity of the AFAQ-Swe, which is essential for determining whether the tool can predict injuries or reinjuries. A recent study, however, reported that the AFAQ showed a good predictive validity for the rehabilitation time needed before a safe return to competition among various athletes [29]. In addition, data from our research group indicate that higher levels on the AFAQ-Swe, measured at the beginning of a soccer season among female elite soccer players, associated with injury problems (non-time-loss injuries) as well as time-loss injuries reported throughout a season of ten months (article in review) [41].

In the rehabilitation of sports injuries, it is common to focus on the biomechanical aspects. Advanced rehabilitation needs to adopt a holistic approach to consider the impact of psychosocial factors. Currently, no valid tool in Swedish exists to measure fear avoidance or fear of reinjury among injured athletes. We propose that utilising the AFAQ-Swe at the start of an athlete’s rehabilitation is beneficial to ensuring a safe return to play. The tool allows the athlete to express concerns about the injury, enabling trainers, coaches, and physiotherapists to address this early in the rehabilitation process, creating an individualised recovery plan and thus a safe return to sport.

## Conclusion

The AFAQ-Swe demonstrates good measurement properties, with strong reliability and validity. The AFAQ-Swe is a concise, user-friendly tool that can be effectively utilised in the Swedish context, particularly among soccer players. However, further research is necessary to evaluate AFAQ’s predictive validity for a safe return to sports.

## Data Availability

Data that support the findings of this study are available from the corresponding author upon reasonable request.
